# Characterization of *PcLEA14*, a Group 5 Late Embryogenesis Abundant Protein Gene from Pear (*Pyrus communis*)

**DOI:** 10.3390/plants9091138

**Published:** 2020-09-03

**Authors:** Tomoki Shibuya, Ryota Itai, Minori Maeda, Hiroyasu Kitashiba, Kanji Isuzugawa, Kazuhisa Kato, Yoshinori Kanayama

**Affiliations:** 1 Faculty of Life and Environmental Science, Shimane University, Matsue 690-8504, Japan; tomoki.s.t.f@gmail.com; 2 Graduate School of Agricultural Science, Tohoku University, Aoba-ku, Sendai 980-8572, Japan; itai1230ok@yahoo.co.jp (R.I.); maedax20@gmail.com (M.M.); hiroyasu.kitashiba.c7@tohoku.ac.jp (H.K.); kazuhisa.kato.d8@tohoku.ac.jp (K.K.); 3 Horticultural Experiment Station, Yamagata Integrated Agricultural Research Center, Sagae, Yamagata 991-0043, Japan; isuzugawak@pref.yamagata.jp

**Keywords:** DREB, LEA14, low-temperature stress, pear, *Pyrus communis*

## Abstract

Fruit trees need to overcome harsh winter climates to ensure perennially; therefore, they are strongly influenced by environmental stress. In the present study, we focused on the pear homolog *PcLEA14* belonging to the unique 5C late embryogenesis abundant (LEA) protein group for which information is limited on fruit trees. PcLEA14 was confirmed to belong to this protein group using phylogenetic tree analysis, and its expression was induced by low-temperature stress. The seasonal fluctuation in its expression was considered to be related to its role in enduring overwinter temperatures, which is particularly important in perennially. Moreover, the function of *PcLEA14* in low-temperature stress tolerance was revealed in transgenic *Arabidopsis*. Subsequently, the pear homolog of dehydration-responsive element-binding protein/C-repeat binding factor1 (DREB1), which is an important transcription factor in low-temperature stress tolerance and is uncharacterized in pear, was analyzed after bioinformatics analysis revealed the presence of DREB cis-regulatory elements in *PcLEA14* and the dormancy-related gene, both of which are also expressed during low temperatures. Among the five *PcDREBs*, *PcDREB1A* and *PcDREB1C* exhibited similar expression patterns to *PcLEA14* whereas the other *PcDREBs* were not expressed in winter, suggesting their different physiological roles. Our findings suggest that the low-temperature tolerance mechanism in overwintering trees is associated with group 5C LEA proteins and DREB1.

## 1. Introduction

Environmental stresses such as extreme temperatures and drought have a significant influence on the growth and yield of crops. Recently, due to concern about the extreme environmental changes arising from global warming [1], an increasing number of studies have focused on the mechanisms underlying herbaceous and woody crops’ tolerance to environmental stresses such as low temperature, drought, and high salinity and how these mechanisms can be utilized to improve crop yields [2,3,4,5,6,7]. It is important that researchers accelerate current studies on crops in this post-genomic era. The expression of genes that enable plants to adapt to environmental stress has been modeled using an herbaceous plant, *Arabidopsis thaliana*. However, particular attention must be paid to trees, especially fruit trees, as they are exposed to high environmental stress in harsh winter climates that need to be overcome to ensure perennially. Therefore, the present study focuses on the molecular mechanisms underlying low-temperature stress tolerance in fruit trees.

Late embryogenesis abundant (LEA) proteins accumulate in large quantities during the late stages of seed embryogenesis and, as predominantly hydrophilic proteins, are believed to be involved in environmental stress response [8]. LEA proteins are classified into seven groups according to their amino acid sequences; however, group 5 proteins are considered atypical LEA proteins because of their comparatively large number of hydrophobic residues and their lack of common sequences and important motifs found in other LEA proteins [8]. Group 5C proteins have a Water Stress and Hypersensitive (WHy) response domain, and their function is expected to differ from that of other LEA proteins [9,10,11]. Although some studies have investigated the stress response of group 5C LEA proteins in herbaceous plants [2,12,13,14], there is limited information available on trees in which a unique functionality of the gene is expected to ensure perennially. This study employs LEA14 to investigate the functionality of these stress proteins in trees.

The dehydration-responsive element-binding protein/C-repeat binding factor (DREB) is the most well-known transcription factor involved in abiotic stress responses. Studies have shown that *DREB* expression is induced by low temperatures, salt stress, as well as drought and that it interacts with NAC transcription factors and activates the expression of downstream genes [15,16]. Compared to herbaceous plants such as *Arabidopsis*, information regarding fruit trees is limited, and to our knowledge, no detailed analysis has been performed on pear trees (*Pyrus communis*). Although omics studies, including ones investigating of stress- and dormancy-related genes, have been conducted on pears [17], they have primarily involved the fruit itself and lack a detailed analysis of individual genes.

DREB1 and DREB2 are subgroups in the DREB family. DREB1 is categorized as a regulator of low-temperature stress responses, whereas DREB2 regulates dehydration and heat-shock responses [15,18]. Given DREB1’s role as a master regulator of cold stress responses [19], it is likely to be involved in inducing the expression of other stress response genes such as *LEA14*. Based on the above, phylogenetic and expression analyses were conducted on *PcDREB1*, the pear homolog of *DREB1*.

In order to understand the molecular mechanisms underlying low-temperature stress tolerance and to determine which genes are useful for enhancing stress tolerance for molecular breeding, we focused on a unique LEA belonging to the group 5C proteins from pears and characterized this protein with *DREB1* homologs. The results in the present study contribute to the understanding of the role of these genes during the winter, which is of principal importance to ensure the perenniality of fruit trees.

## 2. Results

### 2.1. Phylogenetic Analysis and Low-Temperature Induced Expression of the Pear Homolog of LEA14: PcLEA14

**Figure 1:**
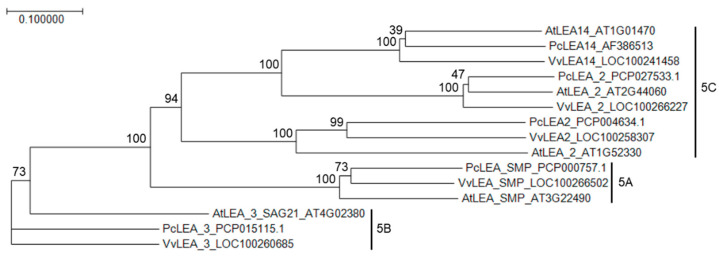
A phylogenetic tree based on the amino acid sequences of PcLEA14 (AF386513) and group 5 late embryogenesis abundant (LEA) proteins from *Arabidopsis*, *P. communis* and *V. vinifera*: The tree was constructed using the neighbor-joining method after sequence alignment using ClustalW2, with Genetyx version 14 (GENETYX CO., Tokyo, Japan). Branch numbers refer to the percentage of replicates that support the branch using the bootstrap method (1000 replicates). The scale bar corresponds to 0.1 amino acid substitutions per residue.

**Figure 2:**
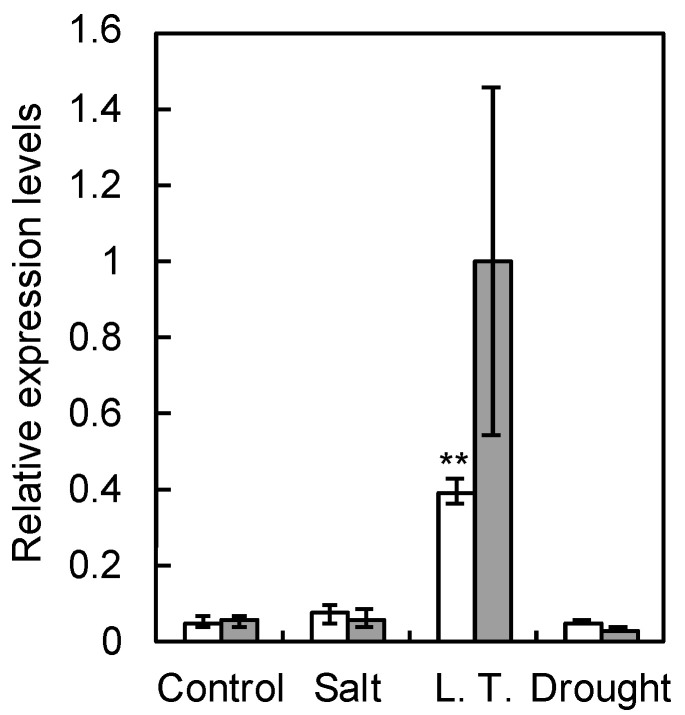
mRNA expression levels of *PcLEA14* in pear leaves under environmental stress conditions for 24 h (white) and 48 h (grey): data shows the relative expression levels normalized against *PcrRNA*. Values indicate the mean ± standard error (*n* = 6). ** Significantly different from control conditions at *p* < 0.01 according to the Dunnett’s test.

A phylogenetic tree based on the amino acid sequences of LEA proteins revealed that the pear homolog of LEA14 is included in the group 5C LEA proteins among groups 5A, 5B, and 5C (Figure 1). Therefore, the homolog was designated as *PcLEA14*. Subsequently, the stress response of *PcLEA14* expression was investigated in pear leaves. The results showed that *PcLEA14* mRNA expression levels increased to approximately eight times that of the control due to low-temperature stress in a period of 24 h, whereas no effects of salt and drought stress on expression levels were observed (Figure 2). 

### 2.2. Seasonal Changes in PcLEA14 Expression

**Figure 3:**
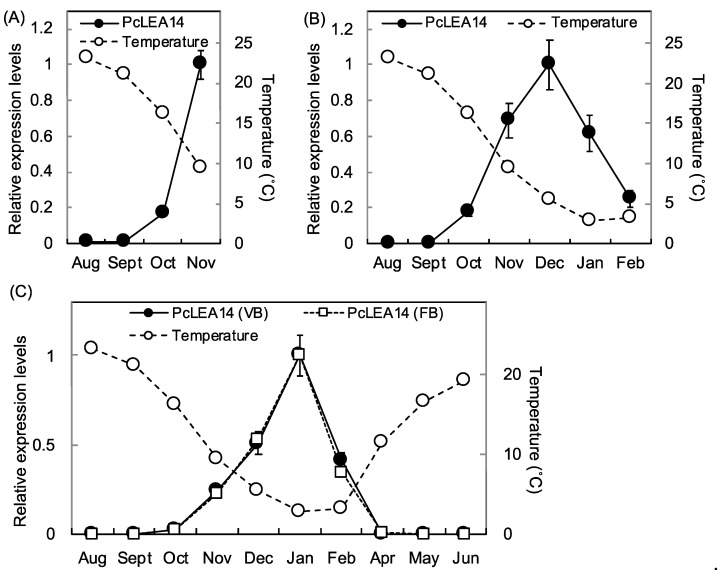
Seasonal changes in mRNA levels of *PcLEA14* in wild-type pear leaves (**A**), stems (**B**), and buds (**C**) at air temperature: each value was determined from three independent biological replicates. Data show the relative expression levels normalized against *PcActin* for leaves and stems and *PcGAPDH* for buds. Values indicate the means ± standard error (*n* = 3). VB and FB indicate vegetative buds and flower buds, respectively (**C**).

The expression of *PcLEA14* was evaluated in mature leaves, current stems, flower buds, and vegetative buds using the pear trees in the orchard. The level of *PcLEA14* mRNA expression in mature leaves was very low in August and September and then increased in October with decreasing temperatures (Figure 3A). No data were obtained for December and later months because of defoliation. The level of *PcLEA14* mRNA expression in current stems also increased from October to December but then decreased after December (Figure 3B). The level of *PcLEA14* mRNA expression in the flower buds and vegetative buds was very low in August and September, but it increased with decreasing temperatures (Figure 3C). These levels peaked in January, then remarkably decreased, and became very low from April onward.

### 2.3. Bioinformatic Analysis of Promoter Sequences

The DRE/CRT motifs, cis-elements targeted by DREB, were searched for in *PcLEA14* to determine candidate genes that may play a role during dormancy and which are expected to be necessary for overwintering. To distinguish the motifs from their promoters, we analyzed the 1500-bp upstream sequences of those genes (Table 1). There were gap regions (NNN…) upstream of PCP000690.1 and PCP022935.1, and hence, we used the sequences between the start codon and the gap region. We identified two DRE/CRT motifs in the *PcLEA14* promoter and either one or two DRE/CRT motifs in the promoters of the candidate genes, except PCP032234.1, PCP029251.1, PCP007373.1, and PCP002349.1. Some of the genes with the DRE/CRT motifs were homologous to genes with functions of interest. MADS-box protein AGL24-like contains the homolog of *DAM/MADS13,* which is known to regulate endodormancy in trees [20,21]. 12-Oxophytodienoate reductase 2-like gene may function in the synthesis of jasmonic acid, involved in dormancy and stress response [22]. Furthermore, protein phosphatase 2C 56-like is an ABI1 homologous gene and could be associated with ABA signaling [23]. On the other hand, the FLOWERING LOCUS T (FT)-interacting protein 1-like is a factor involved in the transport of flowering-inducing factor FT, and the presence of the DRE/CRT motifs in this gene suggests a novel research development regarding the role of DREB.

### 2.4. Seasonal Changes in the Expression of the Pear Homologs of DREB1: PcDREB1s

**Figure 4:**
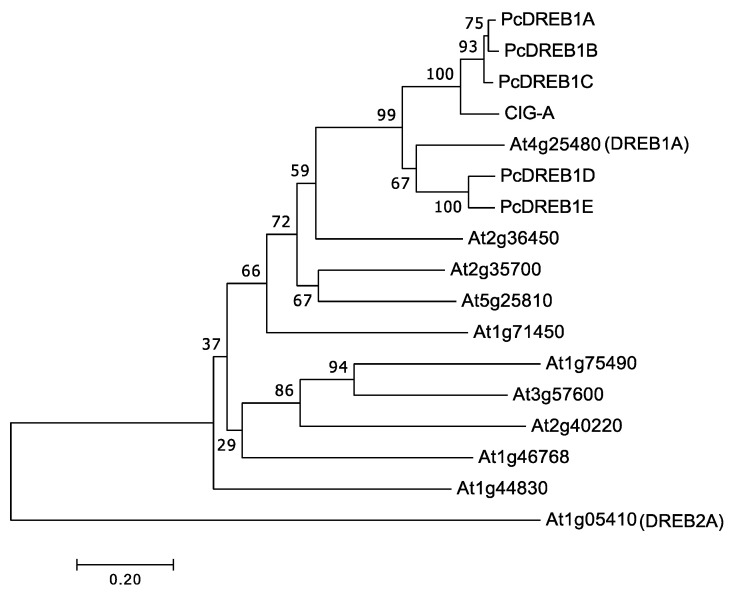
Phylogenetic tree based on the amino acid sequences of PcDREBs and dehydration-responsive element-binding protein/C-repeat binding factor (DREB) proteins from *Arabidopsis*: the tree was constructed using the neighbor-joining method, with MEGA version 7.0. Branch numbers refer to the percentage of replicates that support the branch using the bootstrap method (1000 replicates). The scale bar corresponds to 0.2 amino acid substitutions per residue. The sequences of *Arabidopsis* DREB proteins were obtained from Mizoi et al. [15]. CIG-A accession number: Q8H9A2. Gene names of PcDREBs in the Genome Database for Rosaceae are PCP009299.1 (PcDREB1A), PCP014363.1 (PcDREB1B), PCP011972.1 (PcDREB1C), PCP019717.1 (PcDREB1D), and PCP012284.1 (PcDREB1E).

**Figure 5:**
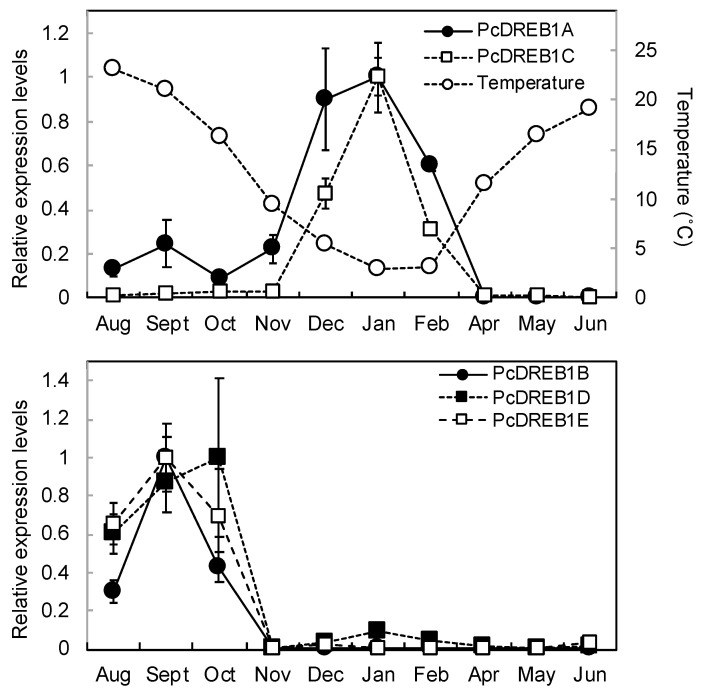
Seasonal changes in the mRNA levels of *PcDREBs* in wildtype pear vegetative buds at air temperature: each value was determined from three independent biological replicates. Data show the relative expression levels normalized against *PcGAPDH*. Values indicate the means ± standard error (*n* = 3).

As indicated by the phylogenetic trees containing the *Arabidopsis* DREB proteins, these PcDREBs belonged to the DREB1 group together with CIG-A (Figure 4). CIG-A is a DREB1 homolog in sweet cherry, which is a rosaceous fruit tree like pear, and its low-temperature tolerance functional analysis has been performed in *Arabidopsis* [32]. Seasonal changes in the expression of these five genes were analyzed in the buds to compare them with *PcLEA14* expression (Figure 5). The level of *PcDREB1A* mRNA expression began to increase in approximately November, peaked from December to January, and markedly decreased from February to April. The level of *PcDREB1C* mRNA expression increased in December, peaked in January, and then markedly decreased from February to April. *PcDREB1B*, *PcDREB1D*, and *PcDREB1E* exhibited similar expression patterns (i.e., their expression was observed from August to October but was very low thereafter).

### 2.5. Stress Tolerance in Arabidopsis Expressing PcLEA14

**Figure 6:**
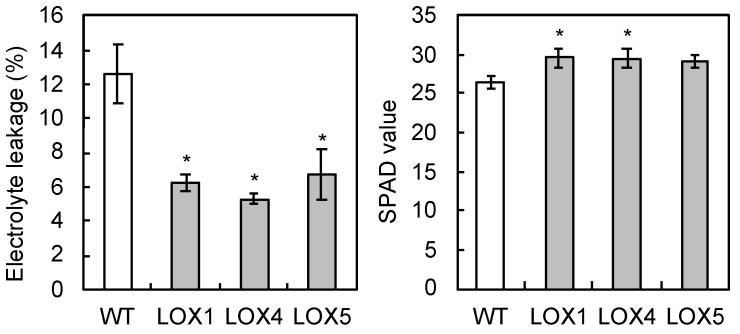
Electrolyte leakage and chlorophyll content (SPAD value) under low-temperature stress conditions at −2 °C in LOX lines of transgenic *Arabidopsis* expressing *PcLEA14*: values indicate the means ± standard error (*n* = 3). * Significantly different from wildtype (WT) at *p* < 0.05 according to Dunnett’s test.

Under the stress condition at −2 °C, electrolyte leakage was lower in *Arabidopsis* expressing *PcLEA14*, LOX lines, than that in the wildtype, and the chlorophyll content was higher in LOX1 and LOX4 than that in the wildtype (Figure 6). Although the expression of *PcLEA14* was confirmed in LOX lines, the mRNA levels of *AtLEA14*, which is the endogenous LEA14 gene in *Arabidopsis*, were not higher in LOX lines than in the wildtype (Supplementary Figure S1). These results suggest that PcLEA14 may contribute to cell survival and maintenance of photosynthetic pigments under low-temperature stress.

## 3. Discussion

The LEA14 pear homolog was confirmed to belong to the group 5C proteins of the LEA family, as shown in the phylogenetic tree analysis, and it was designated as *PcLEA14*. Group 5C proteins have a unique structure [9,10,11], and although their role is unclear, they have been the subject of a small number of studies. An earlier study reported that overexpression of the same gene group in maize, *ZmLEA5C*, improved low-temperature stress tolerance in tobacco plant [2]. Furthermore, in the present study, low temperatures induced the expression of *PcLEA14* and low-temperature stress tolerance in *Arabidopsis* was improved after its overexpression. These results show that one of the roles of the group 5C LEA proteins is a response to low-temperature stress. Conversely, previous studies have reported that, in *Arabidopsis* and sweet potato, AtLEA14 and IbLEA14, which belong to the same group, are involved in both salt and drought stress responses [12,14]. As demonstrated in the expression analysis using pear leaves, the expression of *PcLEA14* was induced only by low temperature. These different reactions suggest a variety of roles for LEA14. Based on these previous reports and the present study, the LEA14 protein itself is considered multifunctional, being effective at both low temperature and drought conditions, and in fact, PcLEA14 may also contribute to drought tolerance in transformed *Arabidopsis* (data not shown). Therefore, it is possible that the diversity of roles may be derived from the fact that the expression pattern differs depending on the species. The *PcLEA14* promoter was found to have CRT/DRE motifs similar to dormancy-related genes in pear, suggesting that *PcLEA14* could be co-expressed with such genes during overwintering.

DREB1 is known as a master regulator of the low-temperature stress response [19], and its homolog plays a role in the induction of the LEA14 homolog in strawberry, belonging to the same rosaceous family as pear [33]. Unlike herbaceous plants, the low-temperature stress response is more important in trees because they are perennial and must endure overwinter conditions; however, there is a lack of information regarding the seasonal expression patterns of *LEA* and *DREB* homologs. In the present study, the expression of *PcLEA14* increased in leaves as temperature decreased in autumn, suggesting a contribution to the low-temperature stress response. Moreover, the expression of *PcLEA14* increased in overwintering stems and buds as the temperature decreased, suggesting its contribution to low-temperature stress tolerance in winter. Among the five PcDREBs, the expression patterns of *PcDREB1A* and *PcDREB1C* were similar to that of *PcLEA14*. In particular, the expression pattern of *PcDREB1C* was most similar to that of *PcLEA14* and PcDREB1C belonged to the same cluster on the phylogenetic tree as CIG-A, which is the DREB1 homolog of sweet cherry of the same rosaceous fruit tree and induced by low temperature [34,35]. *PcLEA14*, in fact, contained DREB cis-regulatory elements in its promoter region, suggesting that PcDREB1A and PcDREB1C are expected to regulate the expression of *PcLEA14.* Moreover, during our studies of sweet cherry DREB1, the introduction of *CIG-A* into pear was confirmed to induce *PcLEA14* expression (data not shown). Conversely, *DREB1B*, *DREB1D*, and *DREB1E* were not expressed in winter but were expressed in summer; therefore, they were not involved in low-temperature stress response and their other possible roles were considered. The distinct difference in the expression patterns found in the *DREB1* subfamily could be associated with their physiological roles. A recent study in *P. pyrifolia* conducted expression analysis of the DREB family gene [36]. Although data was not collected year-round, the expression levels of all genes peaked between October and February and no members with unique expression such as *DREB1B*, *DREB1D*, and *DREB1E* were reported. Because the response to high temperature, drought, and high salinity is related to the wide signal network involving GTPase and rhizosphere bacteria together with DREB and LEA [6,37], it is necessary to analyze *DREB1B*, *DREB1D*, and *DREB1E* from the same broad aspect. 

The level of *PcLEA14* mRNA expression increased with low temperature in the leaves, stems, and buds; however, the expression was not consistently high during low-temperature months. The level of *PcLEA14* mRNA expression decreased in January and February, with the lowest temperature in the stems, and expression also decreased markedly in February, with the lowest temperature in the buds. The mRNA expression levels of *PcDREB1A* and *DREB1C* with *PcLEA14* also decreased in February. These results indicate that the expression levels of *PcDREB1A*, *PcDREB1C*, and *PcLEA14* are regulated by factors other than temperature. Based on data released by the Japan Meteorological Agency, the low-temperature requirement for breaking endodormancy in pear is approximately 1600 h at ≤7.2 °C [38]. During the year in which the expression analysis was performed, the low-temperature requirement reached 1600 h in late January. In fact, the relationship between *LEA* homolog expression and endodormancy has been reported in another rosaceous fruit tree, Japanese apricot [39], although the *LEA* homolog tested is not involved in the group 5C *LEA* proteins. These results suggest that the expression of *PcLEA14* is regulated by both endodormancy and temperature.

## 4. Materials and Methods 

### 4.1. Plant Materials

“La France” pear trees at age 12 years in 2008 were the primary models used for analyzing the expression of *PcLEA14* and its nucleotide sequences. The trees were grown in the Tohoku University (Sendai, Japan) experimental field at 38°16′ N and 140°52′ E. For exploring the seasonal fluctuation in its expression, mature leaves, current stems, and buds were sampled from August to November 2008, from August 2008 to February 2009, and from August 2012 to July 2013, respectively. The samples were randomly obtained from four trees and stored at −80 °C until use. Mature leaves were used for investigating the effect of environmental stress on *PcLEA14* expression. For the transformation experiment in *Arabidopsis*, *PcLEA14* cDNA was cloned into a pBI-OX-GW vector containing a constitutive promoter for plants (Inplanta Innovations Inc., Yokohama, Japan) and the constructed vector was introduced into *A. thaliana* ecotype Columbia using the *Agrobacterium*-mediated transformation method (Inplanta Innovations Inc.). The transgenic plants were selected on media containing kanamycin, and the transgenes were confirmed by PCR. The transgenic *Arabidopsis* lines with *PcLEA14*, LOX1, LOX4, and LOX5 in the T_2_ generation were used with the wildtype Colombia plants as a control. The plants were grown in vermiculite/pearlite (1:1) at 22 °C under a 16 h photoperiod using a growth chamber [40] and used 4 weeks after sowing for experiments.

### 4.2. Sequences Used for Phylogenetic Analysis

The gene *LEA14* of *P. communis* (accession number AF386513) was searched for in the National Center for Biotechnology Information (NCBI). The protein sequences of *Arabidopsis* were used from representative sequences of groups 5A, 5B, and 5C according to the corresponding PFAM number by Battaglia et al. [8]. The pear homologs of group 5 *LEA* genes were searched using BLAST in Genome Database for Rosaceae (GDR). The *Vitis vinifera* homologs of group 5 *LEA* genes were searched by BLAST in NCBI. The pear homologs of *DREB1* were searched using BLAST+ in GDR, and the five genes found in this search were designated as *PcDREB1A*, *1B*, *1C*, *1D*, and *1E*.

### 4.3. Expression Analyses

Total RNA was extracted from pear using the cetyltrimethylammonium bromide method according to Hatsuda et al. [41] and from *Arabidopsis* using the RNeasy Plant Mini Kit (QIAGEN, Hilden, Germany). The removal of genomic DNA and reverse transcription were performed using the Quantiscript Reverse Transcription Kit (QIAGEN) and Rever Tra Ace qPCR RT Master Mix (TOYOBO, Osaka, Japan). Real-time PCR was performed using the QuantiTect SYBR Green PCR Kit (QIAGEN) and THUNDERBIRD SYBR qPCR Mix (TOYOBO) according to Ikeda et al. [42]. The nucleotide sequences of all primers used for the expression analysis are shown in Supplementary Table S1.

### 4.4. Environmental Stress Treatment

The effect of environmental stress on the expression of *PcLEA14* was investigated using detached mature leaves of the wildtype pear “La France” under the conditions of high salinity at 150 mM NaCl, low temperature at 4 °C, and drought. In the control, leaves cut in half horizontally were placed with the cut end facing down in water in tubes and left at 25 °C under dark conditions. The leaves were taken out 24 and 48 h after the start of the treatment and stored at −80 °C; 150 mM NaCl was used instead of water under conditions of high salinity. In the low-temperature stress treatment, the leaves were left at 4 °C instead of 25 °C. No water was added in the drought stress treatment. 

Low-temperature stress tolerance was evaluated at −2 °C for LOX lines of transgenic *Arabidopsis* plants. Stress tolerance was evaluated by measuring electrolyte leakage after stress treatment as described by Kanayama et al. [43] and Feng et al. [44]. After the plants were left at 21 °C for 2 h, the temperature was lowered to −2 °C in 5 h and left for 30 h. Then, the temperature was raised to 21 °C in 5 h followed by cultivation at 21 °C for 2 h. Control plants were cultivated at 21 °C during the treatment. After stress treatment, the plants were grown as described in Section 4.1. for 5 days and used for stress tolerance evaluation.

### 4.5. Bioinformatic Analysis of Promoter Sequences

The upstream 1500-bp *PcLEA14* promoter sequences of the start codon were obtained from *P. communis* Genome v1.0 using BLAST+ on GDR. The homologs of candidate genes associated with dormancy [22] were analyzed using BLAST+ on GDR, and the promoter sequences were obtained as in *PcLEA14*. The promoter sequences were analyzed using the PLACE database [45] for detecting DRE/CRT motifs RCCGAC, RYCGAC, or CCGAC [24,25,26,27,28,29,30,31].

## 5. Conclusion

We identified potential roles of PcLEA14 and PcDREB1s in pear trees using expression analysis, transgenic experiments, and bioinformatic analysis. The DRE/CRT motifs were, in fact, found in *PcLEA14* and in many candidate genes playing a role during dormancy, including genes associated with *DAM/MADS13,* jasmonic acid synthesis, and ABA signaling, which are involved in dormancy and stress response. *PcLEA14* is likely expressed coordinately with these genes and PcDREB1A and C to allow plants to endure harsh winter climates.

## Figures and Tables

**Table 1:** CRT/DRE motif and LTRE motif on the promoter of *PcLEA14* and candidate genes playing a role during dormancy.

Gene Name	Gene ID *P. bretschneideri*	Gene ID *P. communis*	CBFHV ^a^	DRECRT- COREAT ^b^	LTRECORE-ATCOR15 ^c^	Total Counts	Sequence Length (bp)
			Motif: RYCGAC	Motif: RCCGAC	Motif: CCGAC		
PcLEA14	-	PCP029268.1	2	1	1	2	1500
FT-interacting protein 1-like	LOC103967842	PCP000690.1	1	-	-	1	507
MADS-box protein AGL24-like	LOC103964948	PCP022935.1	-	-	1	1	866
MADS-box protein AGL24-like	LOC103964950	PCP022936.1	1	1	1	1	1500
MADS-box protein AGL24-like	LOC103964952	PCP005825.1	2	2	2	2	1500
3-hydroxyacyl-[acyl-carrier-protein] dehydratase FabZ-like	LOC103967963	PCP000700.1	-	-	1	1	1500
chlorophyll a-b binding protein CP24 10A, chloroplastic	LOC103967973	PCP000701.1	2	-	2	4	1500
12-oxophytodienoate reductase 2-like	LOC103967564	PCP032234.1	-	-	-	-	1500
palmitoyl-monogalactosyldiacylglycerol delta-7 desaturase, chloroplastic-like	LOC103954983	PCP032515.1	1	1	1	1	1500
thymidine kinase a	LOC103955051	PCP029243.1	-	-	1	1	1500
chlorophyll a-b binding protein 151, chloroplastic-like	LOC103955064	PCP029251.1	-	-	-	-	1500
cytochrome b6-f complex iron-sulfur subunit, chloroplastic-like	LOC103944475	PCP007373.1	-	-	-	-	1500
protein phosphatase 2C 56-like	LOC103943902	PCP028125.1	1	-	1	2	1500
uncharacterized	LOC103964940	PCP005820.1	1	-	1	2	1500
uncharacterized	LOC103944526	PCP040740.1	1	-	-	1	1500
uncharacterized	LOC103944497	PCP007115.1	1	-	-	1	1500
uncharacterized	LOC103954139	PCP002349.1	-	-	-	-	1500
uncharacterized	LOC103943904	PCP028129.1	2	-	2	4	1500
uncharacterized	LOC103943918	PCP033851.1	1	-	-	1	1500

^a^ [24,25,26], ^b^ [27,28,29], and ^c^ [30,31].

## Supplementary Materials

The following are available online at https://www.mdpi.com/2223-7747/9/9/1138/s1, Table S1: List of primer sequences, Figure S1: mRNA levels of *PcLEA14* and *AtLEA14* in LOX lines of transgenic *Arabidopsis* expressing *PcLEA14*.
