# Supplementary material for: Characterization of PcLEA14, a Group 5 Late Embryogenesis Abundant Protein Gene from Pear (Pyrus communis)
Source: Plants (Basel). 2020 Sep 3;9(9):1138. doi: 10.3390/plants9091138 (PMC7570135; doi:10.3390/plants9091138)
Supplement: Supplementary file 1 [file plants-09-01138-s001.pdf]

Table S1. List of primer sequences

Gene	Sequences of forward (1st line) and reverse (2nd line) primers
AtActin	5'-ACATTGTGCTCAGTGGTGGA-3'
	5'-GAGATCCAACATCTGCTGGAAT-3'
PcActin	5'-GATGACGAAAGAGATTACAGCCTTG-3'
	5'-CCCCGCTTGCTTTGTTTG-3'
PcGAPDH	5'-AGGCTGGAATTGCATTGAAC-3'
	5'-AGATTTAGCAGGTGGACGCTAC-3'
PcLEA14	5'-TTGGTTGTAGGTTTGCGTTTG-3'
	5'-ATGGCTGTAGGGATTGGTCA-3'
AtLEA14	5'-ACTTGGCCAAAGTCTCTGTCAC-3'
	5'-CCGTCATGTCCTTAGCTTTCAG-3'
PcDREB1A	5'-TGGAGCTTCTCAAATTGAATCC-3'
	5'-CAATTGCAAGCCTACACACAAC-3'
PcDREB1B	5'-CTACCGAAACACACACTGAACTC-3'
	5'-GGAGGAATCGGAGATTTGAC-3'
PcDREB1C	5'-GAACTGGGATGATATGGGAAG-3'
	5'-GTGTCCTTAGGGATGATGAATAC-3'
PcDREB1D	5'-AAGAGTCGGATGAGGCTTGTG-3'
	5'-CGCTTCCTCATCCAGAAAATG-3'
PcDREB1E	5'-GTCGGCTTCGGAGACCAG-3'
	5'-GGTGCCGTGTCTCTCTAAACTTC-3'

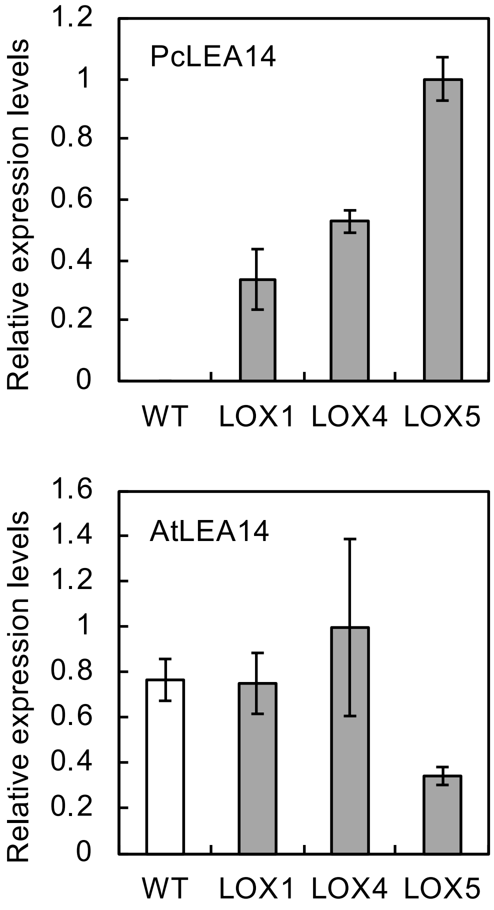

Supplementary Figure S1. mRNA levels of *PcLEA14* and *AtLEA14* in LOX lines of transgenic *Arabidopsis* expressing *PcLEA14*. *AtLEA14* is the endogenous LEA14 gene in *Arabidopsis* and WT indicates wild-type. Each value was determined from three independent biological replicates. Data show the relative expression levels normalized against *AtActin*. Values indicate the means ± standard error (n = 3).
